# Beyond net effects: why fuzzy-set qualitative comparative analysis is the future for modeling social media artrepreneurial success

**DOI:** 10.3389/frma.2025.1719638

**Published:** 2026-01-09

**Authors:** Maria Susan Mathew, Ajimon George

**Affiliations:** Department of Commerce, Marian College Kuttikkanam Autonomous, Kerala, India

**Keywords:** artrepreneurship, configurational causality, digital creativity, fsQCA, fuzzy sets, research metrics, social media

## 1 Introduction

Artrepreneurship is the process of combining an artist's creative abilities with the marketplace for commercial success. It is the practice of bringing artistic endeavors to translate them into money (Hoffmann et al., 2021). Success of present world artrepreneurs is rarely a straight line—artrepreneurs rely significantly on social media, but it cannot be justly regarded that there is a direct relationship between artrepreneurship and social media mastery. The notion of *entrepreneurial success* itself is multifaceted, encompassing financial achievement, personal satisfaction, innovation, and social contribution, yet it has also faced criticism for being conceptually ambiguous and overly focused on economic outcomes (Fisher et al., 2014). A digital artist who masters algorithms and networking may thrive just as much as a talented painter who gains followers through genuine art creation and storytelling. Both succeed- but in entirely different ways.

Most of the available models, however, simply simplify these complex, context-specific results into single-variable measures, or additive measures, as though success can be reduced to net effects of independent factors. With social media-based artrepreneurship, this simplification is what clouds the dynamism of the artistic creativity, the technological involvement and contextual flexibility that, in fact, are the qualities that determine success within the digital creative economies.

These artistic, technological and algorithmic interrelations show why traditional net-effect models such as regression, SEM and correlational approaches are inadequate. They make the assumptions of linearity, independence, and symmetrical causation, and social media artrepreneurship can be characterized as consisting of conjunctural processes, path-dependent processes, and asymmetric processes. This complexity logically demands a set-theoretic approach like fsQCA, which is intended to examine equifinality, causal asymmetry and context-dependent interaction between conditions.

Fuzzy Set Qualitative Comparative Analysis takes prominence here. Often denoted as fsQCA, it provides information on what combinations of causes or conditions make a certain outcome occur, rather than just answering how much one variable has an effect on another variable. The method is particularly applicable to the creative industries where there might be various paths toward success- or as it has been referred to as equifinality. Through the adoption of fsQCA, research in social media artrepreneurship will be able to detect the context-specific, subtle structures that constitute digital creative success, and provide deeper, more accountable modes of explaining success in the arts.

With this topography of causal complexity laid out, it is clear why a framework that is able to model multitrack paths, asymmetrical relations and context-specific interactions is not only desirable but also indispensable. fsQCA explicitly responds to such a methodological requirement by complying with the realities of digital creative labor.

## 2 Theoretical foundations: from entrepreneurial success to fuzzy-set configuration

Success in entrepreneurship as it is captured in this study is conceived to be a multi-factorial construct to the point of going beyond financial measures. It is the personal perception and evaluation of how the criteria that are considered of relevance to the entrepreneur were met. There are many business indicators of success that the entrepreneur may prefer and monetary returns are just one of the possible alternatives. The five major aspects used by the entrepreneur to measure success include the performance of the firm, the relations at work, personal gratification, contribution to the community, and personal monetary gains. This is a wider definition as compared to the conventional perception which tends to lay more emphasis on financial profit or objective economic measures of firm performance (Wach et al., 2016). These multidimensional metrics of success are further disaggregated in the case of social media artrepreneurship since the creators operate within algorithmic visibility, artistic identity, community engagement, and monetization at the same time.

Entrepreneurial success is a multidimensional construct that does not have a universal definition and measurement in the existing literature. This effect can be explained by its pointers and surroundings, including personal and business-level performance. Even the entrepreneurs themselves define success as a combination of personal fulfillment, accomplishment of the business objectives and a positive impact of their businesses. It is marked by the existence of personal as well as macro-level variables implying that it cannot be quantified using only financial or economic quantification (Fisher et al., 2014).

Fuzzy set theory is a mathematical domain that deals with uncertainty, ambiguity or variability in data and reasoning, by classifying objects to a continuum of grades ranging from 0 to 1, rather than the binary membership of ordinary sets. It is a way of describing groups of objects where membership isn't just yes or no but a matter of degree. The ‘membership function' assigns a value between 0 and 1 to each object, indicating how strongly it belongs. Unlike probabilistic approaches, the notion of a fuzzy set is entirely non-statistical (Gupta and Ragade, 1977).

Fuzzy-set Qualitative Comparative Analysis (fsQCA) is an asymmetric data modeling approach that integrates fuzzy sets and fuzzy logic theories with Qualitative Comparative Analysis. It enables the analysis of real-life phenomena by developing simple logical statements that explain distinct combinations of conditions leading to a particular effect. Unlike linear-relationship-based traditional methods that prioritize estimating net effects of individual variables, fsQCA explicitly rejects net-effect logic. Instead, it examines **causal configurations**—combinations of conditions that jointly produce an outcome. Its purpose is not to isolate the impact of a single factor but to understand how multiple conditions co-occur to generate success (Pappas and Woodside, 2021).

The adoption of fsQCA in business and management domain studies a systematic approach used to explore causal complexities inherent within organizations. These complexities are often asymmetric and multilayered. Detailed insights on concepts like organizational and management decision making, employee performance and appraisal, market behaviors and adaptability, etc., can be captured by combining qualitative and quantitative evidence. This is why fsQCA is regarded as a catalyst in studying complexities in the area of business (Ott et al., 2018).

Fuzzy methods have become an integral part of entrepreneurship research since 2016. Studies show a rising trend in the adoption of fuzzy logic in understanding various entrepreneurial dimensions. The most commonly used fuzzy set method in entrepreneurial research is fsQCA, highlighting the main argument, followed by fuzzy Analytic Hierarchy Process (fAHP) and fuzzy Delphi methods. It provides valuable insights on themes like entrepreneurial orientation, innovations, decision making, sustainability and so on Olumekor (2024).

Old school linear approaches fail to capture complex, non-linear configurations of factors that influence entrepreneurial outcomes. This can be mitigated by adopting fsQCA, as it has the inherent ability to handle data asymmetry, equifinality and causal complexity. This makes it suitable for investigating intricate entrepreneurial phenomena, offering understanding toward entrepreneurial best practices (Nikou et al., 2024).

The intensifying application of fuzzy methods, especially the fsQCA in the domain of entrepreneurial psychology, examines complicated, heterogeneous conditions influencing the success of startup ventures. This provides understanding of how different compositions of psychological and contextual variables result in entrepreneurial success (Chu et al., 2022). Similar methodological advances have been demonstrated in recent studies across business management (Douglas et al., 2020) entrepreneurial intention (Kraus et al., 2018) and startup ecosystems (Fiss et al., 2013) showcasing fsQCA's ability to capture causal complexity in entrepreneurial behavior.

Dominant net-effect models, especially regression-based modeling, and SEM assume that causes are independent and additive to effects. This does not fit the essence of artrepreneurial success, where algorithmic exposure, artistic identity and communal co-production all interact in mutually reinforcing ways. This conjunctural causation cannot be narrowed down to the peripheral effect of individual variables.

## 3 Why fsQCA fits the study of social media artrepreneurship

The success of social media artrepreneurial is **configurational** by its very nature. The success of artrepreneurial activities is not the result of a single determinant like posting frequency or follower count. Instead, it is a consequence of the interplay of multiple factors like artistic creativity, content types, interactive mechanisms, algorithm visibility, collaborations, timing and so on. This causal complexity of the conditions leads to equifinality, asymmetry, and conjunctural causation, which are causal issues that fsQCA can uniquely analyze. These multi-path mechanisms cannot be accounted for by linear models since success is assumed to be uniform in its effects, though fsQCA treats success as a result of configurations and therefore is naturally better adapted to digital creative ecosystems.

Artrepreneurial success is **subjective** in nature, i.e. success can take different meanings to different artists. Success can mean income or increased sales for one creator, artistic joy of creation for another and self-actualization for a third. This subjectivity cannot be computed precisely using traditional statistical tools like regression or correlation. Fuzzy logic enables integration of these incomplete and coinciding meanings to co-exist within a single analytical framework.

**Asymmetry** is another important factor emphasized in fsQCA. For instance, the absence of collaborations doesn't necessarily predict failure, but may act as a fuel for artistic autonomy. fsQCA allows us to explain realities of artrepreneurial ventures as they are- nuanced, fluctuating and multidimensional.

## 4 Benefits of methodology compared to net-effect models

All the methodological strengths of fsQCA directly resolve the weaknesses of net-effect models. Where regression isolates variables, their interplay with each other, as a combination, is captured by fsQCA. Where SEM also needs model fit to a single dominant pathway, the multiple and equally valid pathways to success are discovered by fsQCA. The subsequent subsections place each of the advantages of fsQCA in a way as to respond to a particular weakness of the conventional analytical models.

### 4.1 Capturing complex causation

Traditional regression models struggle to detect such combinational causation, which fsQCA captures explicitly. Using logical reasoning, fsQCA examines how various combinations of conditions or events lead to specific outcomes. Instead of just producing coefficients in traditional statistical analysis, fsQCA generates logical equations, or “if-then statements”, which generate a summary of the set of conditions that led to success. This is noteworthy in medium-N studies, which are more common in creative research, when either there are too many cases or items for purely qualitative enquiries or too few for large-scale statistical applications.

### 4.2 Calibration and transparency

Unlike opaque algorithm-driven metrics, fsQCA's calibration foregrounds researcher reasoning. The calibration process of fsQCA is one of its core advantages. From a specific given dataset, researchers can exactly decide what counts as “high” or “low” affiliation. Of course, this needs to be theoretically supported; the choices cannot be solely based on data inference. In a field that is frequently dominated by opaque algorithms and automated measurements, this sense of transparency exhibits reflexivity toward measurement, which can be duly regarded as a progressive move.

### 4.3 Interpretability and theoretical discussion

This contrasts with net-effect outputs that offer limited interpretive depth for creative sectors. fsQCA provides intelligent and interpretable results. Rather than giving a seemingly complex abstract coefficient, say, β = *0.32*, fsQCA offers statements like “*High artistic expression and consistency*→*Success”*. These claims provide valuable insights into established theories, such as effectuation, resource-based views and social capital frameworks; they facilitate productive debates between theory and findings. This interpretability is important to artrepreneurs since knowledge needs to address creative strategy;- authenticity, reach and aesthetic consistency.

### 4.4 Managing multi-path causality and asymmetry

Symmetry assumptions in regression obscure these multidirectional pathways. Since fsQCA examines an outcome's presence or absence independently, it naturally follows an element of asymmetry. It might not always be possible to explain failure by merely reversing the pathways that lead to success. This reflects an important reality of creativity, where the lack of one essential requirement- say, a high number of followers- may be compensated by another, say, aesthetic uniqueness.

### 4.5 Interdisciplinary reach

Where conventional methods force artificial uniformity, fsQCA integrates diverse types of evidence. fsQCA allows cross-disciplinary integration by incorporating quantitative indicators with qualitative ones, say engagement rates with perceived originality. This method is more holistic as it provides a bridge between numerical and narrative evidence, enabling the capture of fruitful nuances on “impact” beyond numbers.

## 5 Implications for research metrics and analytics

Social media artrepreneurship exemplifies the very problem that research metrics also face: complex outcomes being reduced to simple indicators.

### 5.1 Reframing research evaluation through fuzzy and configurational thinking

The paradigm shift toward fuzzy-set and configurational thinking holds implications not only for artrepreneurship research but also for the wider field of research metrics and analytics. Similar tensions exist between both domains- the need to assess complex phenomena by applying indicators that are often crude to capture complexities. In the context of research evaluation, traditionally, citation counts stood as the ultimate indicator of influence. But now more emphasis is given to the recognition that impact is multifaceted, which includes social relevance, novelty in methodology, policy reach and many more. fsQCA offers a conceptual bridge between these debates, showing the importance of relying on combinations of complementary conditions rather than depending on a single indicator.

### 5.2 Moving beyond linear indicators

Traditional analytics assumed that “more is better”—more followers, more likes, more engagement. Yet, creative success is usually a result of a balance of several conditions. For instance, extremely high visibility may reduce authenticity, whereas niche focus might increase community trust. fsQCA gives due recognition to such conditional interdependencies directly.

This approach persuades researchers and scholars to rethink digital metrics themselves- not as distinct numbers but as ingredients which must be combined to produce meaningful insights. This is not just a methodological revamp; it is philosophical, which helps understand performance in digital ecosystems.

### 5.3 Moving toward context-sensitive, responsible assessment

A configurational viewpoint also aligns with the increasing demand for responsible metrics in academia and beyond. Instead of pursuing a single dominant success narrative, fsQCA shows that various forms of success can exist together, with some based on integrity and influence, while others on reach and visibility. It suggests that boosting one indicator may possibly worsen others.

By adopting fuzzy reasoning, institutions and policy makers can develop multi-dimensional evaluation frameworks that honor outcome variabilities. This is particularly significant in the creative economy as value and innovations frequently emerge in unexpected, context-specific ways.

### 5.4 Policy and institutional applications

For organizations that foster creative endeavors like art councils and digital platforms, fsQCA promotes **customized interventions**. Instead of looking for a universal formula for success, they can find clusters of favorable circumstances. The synergy between peer networking and digital literacy instructions, for instance, may be highlighted in one configuration, while algorithmic fluency and novel creativity could be highlighted in another. Such findings can direct training and funding programs that represent the **plurality of creative pathways**, instead of forcing a one-size-fits-all strategy. This is consistent with broader research metric goals—to assess human endeavor in a fair, open and sensitive way.

## 6 Methodological and epistemological challenges

Despite of its benefits, fsQCA has its drawbacks. Just like any other approach that deals with interpretation, fsQCA mostly relies on the judgement and theoretical clarity of the researcher.

### 6.1 Subjectivity and calibration

One recurring concern is **calibration**—deciding what counts as “high” or “low” membership in a fuzzy set. The thresholds that a researcher fixes can have a high impact on the results. Thus, solid theoretical justification, triangulation of data sources and robustness checks are essential. Both a virtue and a weakness, calibration necessitates interpretative discipline and forces openness.

### 6.2 Limited diversity and case sensitivity

Another issue is the **limited diversity problem**—the fact that not all logically possible combinations of conditions are observed in real data. This can constrain generalizability. fsQCA's logic of sufficiency rather than prediction means that findings describe what works in specific contexts, not universal laws. But arguably, this contextualism is an advantage in studying creative domains, where uniqueness is the rule rather than the exception.

### 6.3 Complexity vs. parsimony

fsQCA also requires balance. Results become cumbersome when there are too many conditions; crucial nuances are lost when there are too few. Instead of overcomplicating models, researchers should let theoretical logic direct the addition of conditions.

### 6.4 Rethinking causation

Lastly, a more profound epistemic change is necessary to embrace a configurational framework. Scholars are urged by fsQCA to shift from probabilistic to set-theoretic thinking, from determining the likelihood of an occurrence to determining the circumstances in which it occurs. Those who have been taught in variable-based traditions may find this reorientation unsettling, yet it offers up new conceptual avenues. It pushes us to view causality as compositional and context-bound, which is a viewpoint that feels much more in line with the actual experience of creative work.

### 6.5 Dynamic endogeneity and marginal effects

A recurring critique from quantitative traditions concerns dynamic endogeneity—where successful outcomes reshape the very conditions that produced them. Although the estimation of temporal feedback loops is not the same in fsQCA as it is in econometric models, new extensions like Temporal QCA (TQCA) promise fruitful applications of temporal sequencing in configurational analysis.

The second criticism that has been made is related to the failure to estimate individual variables marginal effects. It is a design aspect and not a limitation: fsQCA does not aim to dispel independent contributions but to expose the way conditions interact. However, scholars can supplement fsQCA with variance-based techniques when they want to get policy-level interpretation.

## 7 Scope for future research

There are several promising directions for extending fsQCA's application to creative industries and beyond:

### 7.1 Longitudinal configurational studies

Most existing fsQCA studies treat time as static, yet creative success is inherently dynamic. **Longitudinal fsQCA** could trace how configurations evolve as platforms, algorithms, and audience preferences change. This would help identify which combinations remain stable and which adapt over time. TQCA or sequence-sensitive calibration rules could be employed to track configuration evolution.

### 7.2 Cross-platform and cross-industry comparisons

A multi-group fsQCA design would allow systematic comparison across platforms. Comparative fsQCA could explore whether the conditions for success on one platform, such as Instagram, differ fundamentally from those on TikTok or YouTube. This would illuminate both **platform-specific** logics and cross-cutting patterns that characterize the broader creative economy.

### 7.3 Cross-cultural configurations

Researchers may need culturally adaptive calibration anchors to reflect differing meanings of authenticity or creativity. Cultural context shapes not only creative practices but also the meaning of success itself. Future studies might compare how fuzzy constructs—authenticity, innovation, or audience intimacy—are calibrated differently across societies. Such research would advance the **globalization of configurational thinking**, enriching both theory and method.

### 7.4 Integration with machine learning and computational methods

Machine learning could identify candidate configurations, which fsQCA then evaluates using set-theoretic logic. The frontier of fsQCA may lie in **hybridization**. Machine learning can help detect hidden configurations or automate calibration, while fsQCA retains interpretability and theoretical grounding. A hybrid approach could merge the strengths of both, producing insights that are data-rich yet conceptually transparent.

### 7.5 Operationalizing “soft indicators”

Finally, future work must refine how we measure fuzzy constructs such as *creative resilience, satisfaction*, or *perceived influence*. These are central to the artrepreneurial experience but difficult to quantify. fsQCA's logic of degrees of membership provides a conceptual toolkit for integrating such soft indicators into empirical analysis. Soft indicators can be operationalized using membership scales developed from qualitative interviews or expert panels.

In short, the future of research on artrepreneurial success may depend on scholars' willingness to experiment—to see methodological pluralism as a strength rather than a threat.

## 8 Conclusion

The growing intersection between art and entrepreneurship demands methods capable of capturing its inherent messiness. Linear models, for all their mathematical precision, struggle to grasp the subtle interplay of creativity, technology, and community that defines success in digital spaces. **Fuzzy-Set Qualitative Comparative Analysis** offers a path forward—one that acknowledges that causes rarely act alone and that success often has more than one origin story.

Moving *beyond net effects* is not merely a statistical preference; it is an epistemological necessity. By embracing configurational thinking, we can better understand not just *whether* creative success occurs but *how* it comes together. For scholars in research metrics and analytics, fsQCA opens the door to a richer, more human way of modeling achievement—one that mirrors the complex, often contradictory realities of creative life itself.

If the aim of research metrics is to measure meaning as well as magnitude, then fsQCA stands as a fitting method for the task. It reminds us that success, like art, resists simplification. Sometimes it is partial, sometimes paradoxical—but always, in one way or another, configurational.

The arguments in this paper put together bring into focus three fundamental propositions: (1) social media art entrepreneurial success is configurational in nature; (2) this complexity cannot be taken into account by traditional net-effect models; and (3) fsQCA offers an empirically flexible and theoretically coherent solution. Set-theoretic approaches should therefore be positioned not as supplementary alternatives but as central methodological tools for future research in creative industries and platform economies.

## References

[B1] Chu C-. C. Zhou Z-. H. Wang X. Wu H. Tian Y. Cai Z. . (2022). The analysis of fuzzy qualitative comparison method and multiple case study of entrepreneurial environment and entrepreneur psychology for startups—evidence from Guangdong-Hong Kong-Macao Greater Bay Area and Southeast Asia. Front. Psychol. 12:751309. doi: 10.3389/fpsyg.2021.751309 35145449 PMC8821655

[B2] Douglas E. J. Shepherd D. A. Prentice C. (2020). Using fuzzy-set qualitative comparative analysis for a finer-grained understanding of entrepreneurship. J. Bus. Ventur. 35:105970. doi: 10.1016/j.jbusvent.2019.105970

[B3] Fisher R. Maritz A. Lobo A. (2014). Evaluating entrepreneurs' perception of success: development of a measurement scale. Int. J. Entrep. Behav. Res. 20, 478–492. doi: 10.1108/IJEBR-10-2013-0157

[B4] Fiss P. C. Marx A. Cambré B. (2013). “Chapter 1 Configurational Theory and Methods in Organizational Research: Introduction,” in Research in the Sociology of Organizations (Vol. 38), eds. P. C. Fiss, B. Cambré, and A. Marx (Leeds: Emerald Group Publishing Limited), 1–22.

[B5] Gupta M. M. Ragade R. K. (1977). Fuzzy set theory and its applications: a survey. IFAC Proc. 10, 247–259. doi: 10.1016/B978-0-08-022010-9.50038-4

[B6] Hoffmann R. Coate B. Chuah S-. H. Arenius P. (2021). What makes an artrepreneur?: An exploratory study of artrepreneurial passion, personality and artistry. J. Cult. Econ. 45, 557–576. doi: 10.1007/s10824-021-09413-8

[B7] Kraus S. Ribeiro-Soriano D. Schüssler M. (2018). Fuzzy-set qualitative comparative analysis (fsQCA) in entrepreneurship and innovation research—the rise of a method. Int. Entrep. Manag. J. 14, 15–33. doi: 10.1007/s11365-017-0461-8

[B8] Nikou S. Mezei J. Liguori E. W. El Tarabishy A. (2024). FsQCA in entrepreneurship research: opportunities and best practices. J. Small Bus. Manag. 62, 1531–1548. doi: 10.1080/00472778.2022.2147190

[B9] Olumekor M. (2024). Fuzzy methods in entrepreneurship research. J. Entrep. 33, 365–392. doi: 10.1177/09713557241255415

[B10] Ott U. F. Sinkovics R. R. Hoque S. F. (2018). “Advances in Qualitative Comparative Analysis (QCA): application of fuzzy set in business and management research,” in The SAGE Handbook of Qualitative Business and Management Research Methods: Methods and Challenges, eds. C. Cassell, A. Cunliffe, and G. Grandy (London, UK: SAGE Publications Ltd.), 414–430.

[B11] Pappas I. O. Woodside A. G. (2021). Fuzzy-set Qualitative Comparative Analysis (fsQCA): guidelines for research practice in information systems and marketing. Int. J. Inf. Manage. 58:102310. doi: 10.1016/j.ijinfomgt.2021.102310

[B12] Wach D. Stephan U. Gorgievski M. (2016). More than money: developing an integrative multi-factorial measure of entrepreneurial success. Int. Small Bus. J. Res. Entrep. 34, 1098–1121. doi: 10.1177/0266242615608469

